# Experienced males recognise and avoid mating with non-virgin females in the western flower thrips

**DOI:** 10.1371/journal.pone.0224115

**Published:** 2019-10-17

**Authors:** Adeyemi O. Akinyemi, William D. J. Kirk

**Affiliations:** School of Life Sciences, Keele University, Newcastle-under-Lyme, Staffordshire, United Kingdom; Chinese Academy of Agricultural Sciences Institute of Plant Protection, CHINA

## Abstract

The western flower thrips *Frankliniella occidentalis* (Thysanoptera: Thripidae) is a major insect pest on a wide range of crops throughout the world. There are several unexplained aspects of the mating behaviour, particularly in relation to male-male competition and mate choice. The objectives of the study were to test whether males can detect the mating status of females and whether males avoid mating with previously mated females. Experiments involved either ‘experienced’ adults taken from a laboratory culture, which had been exposed to high densities of thrips, or virgin adults reared individually. Experienced males mated readily with virgin females, but avoided mating with experienced females. Virgin males showed much less discrimination between females. Avoidance of mating with experienced females is likely to be widespread because it occurred in populations from both the United Kingdom and Kenya. Experienced males also mated with dead virgin females, but avoided mating with dead experienced females, which ruled out the possibility that behavioural differences between the females were responsible. To test whether males could detect whether or not females had mated, virgin females of the same age from the same cohort were either mated once or not mated. Experienced males mated with the dead females that were virgin and avoided mating with the dead females that differed only in that they had mated once shortly before. This showed that males recognise whether or not a female has mated and avoid mating with previously mated females. This avoidance by males suggests that mated females are not usually subjected to high levels of male harassment. The most likely explanation of the avoidance is that males produce an antiaphrodisiac pheromone that is applied to females during mating.

## Introduction

The western flower thrips *Frankliniella occidentalis* (Pergande) (Thysanoptera: Thripidae) is a major invasive crop pest that lives on flowers and leaves of many plant species [1,2]. It was originally limited to western parts of North America, but has now spread to become a worldwide crop pest [1]. The mating behaviour involves male aggregations, typically on flowers, in which males produce an aggregation pheromone that attracts both males and females [3,4]. The behaviour within aggregations has been described in detail [5–8]. Females fly to the aggregations, mate and then depart. Males often fight each other within the aggregations, but the advantage of this is unclear, because females that land in aggregations appear to mate with the first male they encounter [6,7]. Thus, males that win fights do not appear to gain any advantage. It has been proposed that males may gain a mating advantage by fighting to defend areas on flowers where females are more likely to land [6], but this has not been tested. Although male aggregations resemble leks, there is no evidence so far that males gain a mating advantage by competing or that females choose between males, which are usual features of classical lek mating systems [9]. Questions remain about the role of competition between males and the extent of mate choice by males and females within aggregations.

Male-male competition can also occur through sperm competition if more than one male mates with the same female. If the first male to mate has a strong advantage, subsequent males can avoid a competitive disadvantage and waste of resources by not mating with previously mated females.

Previous observations in the laboratory found that females mated once and then rejected subsequent males by flipping the abdomen up and down [8]. Rejections occurred immediately after mating and also after several days, but some females mated again after 15 days. In the field, some females (14%) mated with at least two males and even as many as six, but these repeat copulations were shorter and were interpreted as ineffective because copulations of similar duration by virgin females in the laboratory produced no female offspring [6,8]. In contrast, some females were ignored by males within aggregations [7]. It was concluded that females are ‘relatively monandrous’ and are in control of mating opportunities for the males [8].

Evidence so far for *F*. *occidentalis* suggests that males usually attempt to mate with previously mated females, since mated females appear to be exposed to repeated harassment by males attempting to mate [8]. In other thrips species, male harassment has been shown to be costly to females by reducing their fecundity and longevity [10,11]. The extent to which males attempt to mate with non-virgin females is therefore important not just in relation to male-male competition, but because it can affect the reproductive rate of females.

The observations of mating behaviour in *F*. *occidentalis* raise several questions. If repeat matings by females are ineffective, why do some males make repeated attempts to mate with mated females? Why do males mate readily with virgin and mated females in the laboratory, but ignore some females in aggregations?

Mating behaviour has been particularly studied in two other species of thrips, which appear to show contrasting behaviours. In the poinsettia thrips *Echinothrips americanus* Morgan (Thripidae), which lives on leaves of a wide range of plant species [12], there is strong evidence that females mate only once [10,13]. Males mark females with an antiaphrodisiac pheromone (dimethyl adipate and dimethyl glutarate) during mating, which deters further mating attempts [13]. However, in the onion thrips *Thrips tabaci* Lindeman (Thripidae), which also lives on leaves and flowers of many plant species [14], females mate multiple times and are subject to high rates of male harassment [11].

Our preliminary observations of mating in *F*. *occidentalis* suggested that males were often unwilling to mate with females. Since these observations were at variance with other reported findings [6,8], we studied male mating behaviour in *F*. *occidentalis* in two different countries. We specifically asked the following questions. Can males detect the mating status of females? Do males avoid mating with previously mated females?

## Materials and methods

### Thrips rearing

*Frankliniella occidentalis* was reared at Keele University in the United Kingdom (UK) and at the International Centre of Insect Physiology and Ecology (*icipe*) in Nairobi, Kenya. Those in the UK were maintained on chrysanthemum plants (*Dendranthema grandiflorum* (Ramat.) Kitam.) in cages at 26 ± 2°C and L16:D8. The culture was obtained from commercial greenhouses in the UK. Cohorts of virgin thrips were reared on clean French bean pods (*Phaseolus vulgaris* L.) with pine pollen (*Pinus sylvestris* L.). Mature larvae or pupae were removed from the pods and placed individually in modified microcentrifuge tubes (1.5 ml) to ensure their virginity as adults. A hole of about 4 mm diameter was melted into the lid of the microcentrifuge tube and a small piece of tissue was sealed under the lid to allow ventilation while preventing escapes. A small portion of bean pod with pine pollen was placed in the tube as a food source. The tubes were monitored daily for adult emergence to ensure adults were of the required age. In Kenya, *F*. *occidentalis* was maintained in the same way, except that we used clean French bean pods in glass jars at 25 ± 2°C and L12:D12. The culture was obtained from a French bean crop in Kenya. Individual virgins were reared in the same way as above, but without pine pollen. On rare occasions, other thrips species entered the cultures on plant material, but these were visually distinct. The identity of *F*. *occidentalis* could be confirmed during the experiments and distinguished from potential contaminant species by a combination of body colour and pattern, ocellar colour (red) and uninterrupted wing-vein setae.

### Mating behaviour observations

To observe mating behaviour, an adult male and an adult female were placed together in a mating arena (5 mm diameter, height 1.5 mm) made from part of a sheet of toughened dental modelling wax [5]. The arena was cut out of the wax with a cork borer and was sandwiched between a glass microscope slide and a glass cover slip. Thrips were transferred carefully with a cleaned fine brush. Each pair was observed for 10 min or until copulation had finished and the pair had separated. In the UK, the behaviour was measured from high-definition video filmed on a Canon EOS 70D digital SLR camera mounted on a stereoscopic microscope with illumination from a fibre-optics light source. All experiments were performed at 25 ± 1°C. A new arena was used for each female.

In Kenya, thrips were observed in the same way, except that the behaviour was observed directly down a stereoscopic microscope with an LED light source at 26 ± 1°C and not recorded.

The mating behaviours, which occurred in sequence, were: (1) ‘contacted’ (first physical contact, usually occurring in the form of male antennation of the female body); (2) ‘climbed’ (male climbed on the back of the female and aligned its body to face the same direction); (3) ‘bent abdomen’ (male curled its abdomen beneath that of the female while on the back of the female); and (4) ‘copulated’ (prolonged contact between the end of the male abdomen and the end of the female abdomen).

The virgin adults had not been exposed to any other adult thrips and were considered as ‘inexperienced’, whereas the adults from the culture had been exposed to high densities of males and females and were considered to be ‘experienced’, following the terminology of Dukas [15,16]. The inexperienced females were all 3–5 days old, whereas the experienced adults were of unknown age, but likely to be older on average because adults live several weeks [2]. Measurements of the copulation durations of a sample of these experienced females showed that they had nearly all mated at least once, because copulations are shorter for previously mated females than for virgin females [17]. We used experienced adults in many of our experiments because these were sampled from the culture and so represent the general responses that males and females encounter in a population.

### Effects of male and female experience on mating behaviour

We used mating arenas, as described above, to observe the mating behaviour and test mate choice. Experiment 1 compared the mating behaviour of live virgin males with either a live virgin female (3–5 days old) or a live experienced female, and was conducted in the UK with thrips from the UK. Experiment 2 was the same as experiment 1, but used live experienced males. Experiment 3 was a repeat of experiment 2, except that the experiment was conducted in Kenya with a population of thrips from Kenya.

### Mating behaviour of males with dead females

To test whether female behaviour affected male choice, we investigated the behaviour of males with dead females. Male thrips can still copulate with females that are dead [18], and this reveals male responses without any behavioural effects or cues from the female. The females were placed individually in a microcentrifuge tube and frozen for 40 min in a freezer to kill them and then defrosted for a minimum of 10 min. An individual dead female was placed in the arena and a male was then introduced. We used mating arenas, as described above, to record the mating behaviour and test mate choice. Experiment 4 compared the mating behaviour of live experienced males with either a dead virgin female (3–5 days old) or a dead experienced female.

### Mating behaviour of males with virgin and mated females

To test whether mating by the female was the factor that allowed males to distinguish virgin females from experienced females, we controlled for female age, exposure to other thrips and host plant by taking a group of virgin females of the same age from the same cohort that were then either placed in an arena and mated once with a virgin male (3–5 days old) or placed in an arena on their own and not mated, before being frozen. The time between mating and being frozen was 45 to 150 min and the time between mating and the bioassay itself was 95 to 200 min. A new arena was used for each observation. Experiment 5 compared the mating behaviour of live virgin males (3–5 days old) with either a dead virgin female (3–5 days old) or a dead mated female (3–5 days old). Experiment 6 was the same as experiment 5, but used live experienced males. Experiments 4–6 were conducted in the UK with thrips from the UK.

In all experiments, the replicates of the two treatments were completed on the same day or days and arranged so that treatments were not confounded with time.

### Photography

Mating thrips were photographed down a Huvitz HSZ-ZB700 stereomicroscope (Huvitz Corp., South Korea) with a Moticam 1080 Full HD camera (Motic Hong Kong Ltd). Video of thrips mating (S1 Video) was filmed on a Canon EOS 70D digital SLR camera mounted on a stereoscopic microscope with illumination from a fibre-optics light source.

### Data analysis

The frequencies of behaviours were compared between treatments with a Fisher’s exact test for a 2 x 2 contingency table with the frequencies of pairs that did or did not exhibit the behaviour as one factor and whether the females were virgin or experienced/mated as the other. The same test was used to compare the avoidance of experienced/mated females between virgin and experienced males, with the frequencies of pairs that did or did not copulate as one factor and whether the males were virgin or experienced as the other. The tests were carried out with R v3.5.3 [19].

## Results

### Mating behaviour observations

**Fig 1:**
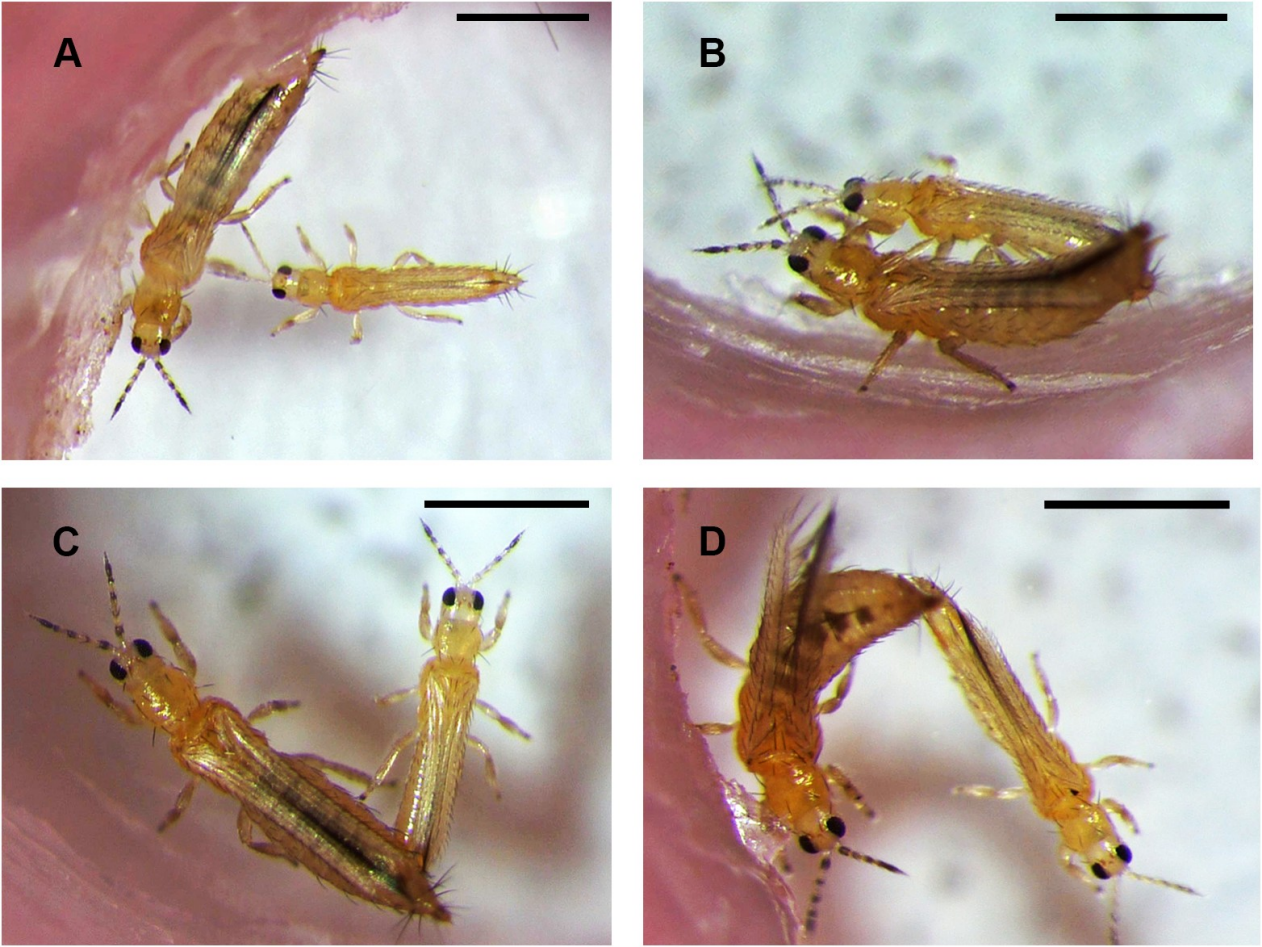
Photographs of the mating behaviour of an experienced male with a virgin female. In each photograph, the male is the smaller of the two. (A) Male antennating a female at first contact. (B) Male and female copulating, with the male antennating and stroking the female with a mid leg. (C) Male and female near the end of copulation in the V-shape position. (D) Male walking away at the end of copulation and pulling at the female abdomen until they separate. Scale bar = 500 μm.

The behaviour of male and female pairs that copulated generally followed the sequence described by Terry & Schneider [8]. During observations of virgin or experienced males mating with virgin females, a male typically approached the female from the front or side and antennated her briefly (Fig 1A) before climbing on the back of the female and aligning his body so that male and female faced in the same direction. The male then antennated the female’s antennae and bent his abdomen underneath that of the female. After attachment was completed by inserting the male aedeagus into the female vagina, the male continued antennating the female antennae. About 4 s after attachment, the male began to stroke the dorsal surface of the female thorax and abdomen with a mid leg (Fig 1B). The female then became calm with little or no movement and the male stroking continued for at least half the copulation period, whereas antennation stopped much earlier, about a quarter of the way through. After the end of stroking, the male and female sometimes moved into a characteristic V-shape position (Fig 1C). Copulation usually ended with the male walking away and pulling the tips of the abdomens apart (Fig 1D).

The initial contact, climbing and bending the abdomen usually took place in rapid succession in about 15 s, sometimes much less, whereas the duration of copulation, with male stroking and the female remaining still, lasted 226 ± 14 s (*n* = 16) for virgin males with virgin females and 208 ± 16 s (*n* = 15) for experienced males with virgin females.

Receptive virgin females usually bent the tip of the abdomen up as the male climbed onto the female (at 00 m 06 s in S1 Video), which appeared to make it easier for the male to secure attachment. This behaviour has not been described before.

When an experienced male and an experience female pair made contact but did not copulate, the male walked away either after the initial antennation or after climbing on the female, but before bending the abdomen. Although these are distinct stages of male behaviour, they can happen in rapid succession and may be only a few seconds apart.

### Effects of male and female experience on mating behaviour

Experiments 1 and 2. Both live virgin males and live experienced males differed in their mating behaviour towards live virgin females and live experienced females (Table 1). Both types of male mated readily with virgin females (100% and 94% copulating), but the experienced males were more discriminating and avoided copulating with experienced females to a greater extent (6% copulating) than the virgin males (69% copulating) (Fisher’s exact test, *P* < 0.001). The experienced males also avoided mating at an earlier stage in the behavioural sequence. The difference in behaviour was statistically significant immediately after contact and before the climbing stage for experienced males (Fisher’s exact test, *P* < 0.001), but only after bending the abdomen for virgin males (Fisher’s exact test, *P* = 0.04) (Table 1). Most experienced males walked away from experienced females after contact or after climbing and there was little or no abdominal flipping by the females. Experienced males showed much greater discrimination than virgin males, so their responses were investigated further.

**Table 1:** The percentage of male-female pairs that exhibited each mating behaviour in the sequence of mating behaviours for live virgin males (experiment 1) or live experienced males (experiment 2) with either live virgin females or live experienced females in the UK.

Behaviour	Experiment 1 with live virgin males (UK)			Experiment 2 with live experienced males (UK)		
	Live virgin females (*n* = 16)	Live experienced females (*n* = 16)	*P* ^a^	Live virgin females (*n* = 16)	Live experienced females (*n* = 16)	*P* ^a^
Contacted	100	100	1.00	94	94	1.00
Climbed	100	100	1.00	94	27^b^	<0.001
Bent abdomen	100	94	1.00	94	19	<0.001
Copulated	100	69	0.04	94	6	<0.001

‘Experienced males’ and ‘experienced females’ had been exposed to high densities of males and females.

^a^ *P*-values were calculated using Fisher’s exact test for a 2 x 2 contingency table with the frequencies of pairs that did or did not exhibit the behaviour against whether the live female was virgin or experienced.

^b^ The observation of whether the behaviour occurred was obscured briefly for one pair, so this was omitted from the analysis for that behaviour.

Experiment 3. The avoidance of mating with experienced females by experienced males (experiment 2) was at variance with other observations (see Introduction), so we tested whether the behaviour was reproducible in a very different population on French beans in Kenya. The difference in behaviour towards virgin females and experienced females was statistically significant immediately after contact and before the climbing stage (Fisher’s exact test, *P* < 0.001) (Table 2). The result was very similar to that obtained in the UK.

**Table 2:** The percentage of male-female pairs that exhibited each mating behaviour in the sequence of mating behaviours for live experienced males with either live virgin females or live experienced females in Kenya.

Behaviour	Experiment 3 with live experienced males (Kenya)		
	Live virgin females (*n* = 42)	Live experienced females (*n* = 42)	*P* ^a^
Contacted	100	100	1.00
Climbed	100	70^b^	<0.001
Bent abdomen	98	42^b^	<0.001
Copulated	92^b^	2	<0.001

‘Experienced males’ and ‘experienced females’ had been exposed to high densities of males and females.

^a^ *P*-values were calculated using Fisher’s exact test for a 2 x 2 contingency table with the frequencies of pairs that did or did not exhibit the behaviour against whether the live female was virgin or experienced.

^b^ The observation of whether the behaviour occurred was obscured briefly for two pairs, so these were omitted from the analysis for that behaviour.

### Mating behaviour of males with dead females

Experiment 4. To test whether male discrimination between virgin and experienced females was the result of behavioural differences between the females, such as resistance by experienced females, we tested the male response to dead females. Live experienced males differed in their behaviour towards dead virgin females and dead experienced females (Table 3). The difference was statistically significant (Fisher’s exact test, *P* < 0.001) after climbing and before the abdomen bending stage. The males mostly walked away from dead experienced females after contact or after climbing.

**Table 3:** The percentage of male-female pairs that exhibited each mating behaviour in the sequence of mating behaviours for live experienced males with either dead virgin females or dead experienced females.

Behaviour	Experiment 4 with live experienced males (UK)		
	Dead virgin females (*n* = 30)	Dead experienced females (*n* = 30)	*P* ^a^
Contacted	77	73	1.00
Climbed	70	43	0.07
Bent abdomen	60	14 ^b^	<0.001
Copulated	57	0	<0.001

‘Experienced males’ and ‘experienced females’ had been exposed to high densities of males and females.

^a^ *P*-values were calculated using Fisher’s exact test for a 2 x 2 contingency table with the frequencies of pairs that did or did not exhibit the behaviour against whether the dead female was virgin or experienced.

^b^ The observation of whether the behaviour occurred was obscured briefly for one pair, so the pair was omitted from the analysis for that behaviour.

### Mating behaviour of males with virgin and mated females

Experiments 5 and 6. To test whether mating by the female was the factor that allowed males to distinguish virgin females from experienced females, we compared responses to females of the same age and from the same cohort that were either virgin or had mated once. We used both virgin males (experiment 5) and experienced males (experiment 6) to allow a further test of whether the two types of male differed in their discrimination between females. Live virgin males showed no significant difference in the proportion copulating between dead virgin and dead mated females (Fisher’s exact test, *P* = 0.37) (Table 4), whereas live experienced males differed in their behaviour towards dead virgin females and dead mated females (Table 4). As in experiments 2, 3 and 4 with experienced males, the difference was statistically significant (Fisher’s exact test, *P* = 0.001) before the abdomen bending stage. Most experienced males walked away from dead mated females after climbing. The experienced males were more discriminating and avoided copulating with mated females to a greater extent (0% copulating) than the virgin males (40% copulating) (Fisher’s exact test, *P* = 0.04).

**Table 4:** The percentage of male-female pairs that exhibited each mating behaviour in the sequence of mating behaviours for live virgin males (experiment 5) or live experienced males (experiment 6) with either dead virgin females or dead mated females.

Behaviour	Experiment 5 with live virgin males (UK)			Experiment 6 with live experienced males (UK)		
	Dead virgin females (*n* = 10)	Dead mated females (*n* = 10)	*P* ^a^	Dead virgin females (*n* = 11)	Dead mated females (*n* = 11)	*P* ^a^
Contacted	100	80	0.47	91	73	0.59
Climbed	90	80	1.00	82	45	0.18
Bent abdomen	80	70	1.00	73	0	0.001
Copulated	70	40	0.37	55	0	0.01

‘Experienced males’ and ‘experienced females’ had been exposed to high densities of males and females. ‘Mated females’ were the same age and from the same cohort as the ‘virgin females’ in the same experiment, but had been mated once with a virgin male.

^a^ *P*-values were calculated using Fisher’s exact test for a 2 x 2 contingency table with the frequencies of pairs that did or did not exhibit the behaviour against whether the dead female was virgin or mated.

## Discussion

Experienced males showed more discrimination between females than virgin males. This phenomenon is also known in fruit flies, where inexperienced males mate indiscriminately, but learn to discriminate through courtship experience with females [16]. The greater discrimination in experienced male thrips compared with virgin males may result from learning acquired from exposure to females over several successful and unsuccessful matings. However, there are alternative explanations, such as a change in choosiness with age [20]. Virgin males sometimes copulate with a female and then return and attempt to copulate again [8], so learning does not appear to be an immediate effect of one successful mating. The possibility that thrips learn from mating experience deserves further study.

In the four experiments with experienced males (2, 3, 4, 6), the proportion of pairs that copulated differed according to the type of female. There were few or no copulations with experienced females. Observations provided no evidence that copulations were abandoned as a result of the behaviour of the experienced females, because both types of female (virgin and experienced) appeared to behave in the same way. In nearly all cases, experienced males walked away from experienced females without encountering any female flipping. In experiments 4 and 6, the females were dead, so female behaviour could not have played any role. It is therefore likely that it was the males that decided whether or not to proceed with mating in all four experiments.

The male avoidance of mating with experienced females observed in the UK (experiment 2) appeared to be different from the previously reported observations in Arizona, USA, in which males repeatedly attempted to mate with non-virgin females [8]. In case this was due to local genetic or environmental differences between the two thrips populations, we repeated the experiment with a population in Kenya (experiment 3) and obtained a similar result. This showed that the observed behaviour was reproducible elsewhere and so likely to be widespread. The difference in male behaviour may instead be due to the status of the males that were used, because experienced males are more discriminating in mate choice than virgin males. Caution should be used in drawing general conclusions about mating behaviour from bioassays with virgins [21].

Experienced females had been previously exposed to many males and females because of the high density of thrips in the culture, and this would be similar to what would be experienced in the field, because the species is abundant in flowers in its native area [22], as well as in flowers of infested crops elsewhere [23]. However, this means that they differ in several ways from virgin females reared in isolation. The critical difference between virgin females and experienced females used in experiments 1 to 4 could be mating status, but could also be age or exposure to other adults or the host plant species. Experiments 5 and 6 were designed to separate out the effect of female mating from these other factors. They showed that experienced males recognise and avoid mating with females that have mated once, compared with virgin females of the same age from the same cohort. Since the differences in behaviour of the experienced males towards the virgin and mated females in experiment 6 were similar to those seen in experiments 2 to 4, it is likely that female mating status also explains those results. Experienced males were sampled from the culture, so they represent the males to which females are generally exposed. These males avoided mating with mated females and mostly walked away after first contact or shortly afterwards, which suggests that mated females in a population will not be subject to repeated male harassment.

The initial contact between males and females usually involved male antennation of part of the female body. Since this is the stage at which experienced males were distinguishing virgin and experienced females, it is likely that the relevant cues are on the surface of the female’s cuticle. Males also distinguished females after climbing, which is also a time when males are antennating the cuticle of the female. The evidence points strongly to a male-produced antiaphrodisiac pheromone that is applied to females during mating and deters subsequent males from mating with that female, as has been found and identified in *E*. *americanus* [13]. The use of such a pheromone in *F*. *occidentalis* could explain the field observation that some females walked around within male aggregations with few males attempting to mate with them [7]. The pheromone could be applied by the male when stroking during copulation. A cuticular hydrocarbon, 7-methyltricosane, that is predominant on adult males has already been identified as a male-produced contact pheromone in *F*. *occidentalis* [24] and could act as an antiaphrodisiac pheromone, but this has not yet been tested. The 7-methyltricosane molecule is chiral and it is not known whether males produce (*R*)-7-methyltricosane or (*S*)-7-methyltricosane or both [24]. A similar cuticular hydrocarbon, 7-tricosene, is predominant on males of the fruit fly *Drosophila melanogaster* and is transferred to the female cuticle during courtship; it then inhibits subsequent male courtship [25,26].

In experiment 6, males distinguished females that had mated 95 to 200 min previously. It is not known how long previously the females had mated in experiments 1 to 4, but it is likely to be longer than that on average. Experiments are needed to test for how long females are avoided by males after the females have mated.

An alternative explanation of the avoidance of mated females is that the females produce an antiaphrodisiac pheromone on the surface of their own cuticle following mating. This is considered less likely because females are unlikely to have had enough time to change the compounds over their cuticle in the period of only 45 to 200 min before they were frozen and also because a male-produced antiaphrodisiac pheromone is already known from *E*. *americanus*, which is in the same sub-family [13].

We have shown that males recognise and avoid mating with non-virgin females in the western flower thrips. This is presumably advantageous to males in a monandrous mating system because it avoids the costs of courting an unreceptive female [27]. It probably also means that females inseminated by a male receive less harassment by other males and so produce more offspring. In the Introduction, we raised the question of why males should attempt to mate with mated females if the matings were ineffective. The answer appears to be that in fact most males avoid such matings.

## Supporting information

S1 File Spreadsheet with raw data from experiments 1–6. (XLSX)

S1 Video Video of an experienced male mating with a virgin female of *Frankliniella occidentalis*. (MP4)
